# A retrospective analysis of Victorian and South Australian clinical registries for prostate cancer: trends in clinical presentation and management of the disease

**DOI:** 10.1186/s12885-016-2655-9

**Published:** 2016-08-05

**Authors:** Rasa Ruseckaite, Kerri Beckmann, Michael O’Callaghan, David Roder, Kim Moretti, Jeremy Millar, Sue Evans

**Affiliations:** 1 Department of Epidemiology and Preventive Medicine, Monash University, Melbourne, VIC Australia; 2 Centre for Population Health Research, Sansom Institute for Health Research, University of South Australia, Adelaide, SA Australia; 3 South Australian Prostate Cancer Clinical Outcomes Collaborative, Department of Urology, Repatriation General Hospital, Adelaide, SA Australia; 4 Flinders Centre for Innovation in Cancer, Flinders University, Adelaide, SA Australia; 5 Freemasons Foundation Centre for Men’s Health and Discipline of Medicine, University of Adelaide, Adelaide, SA Australia; 6 Radiation Oncology, Alfred Health, Melbourne, VIC Australia

**Keywords:** Prostate cancer, Clinical registry, Trends, Treatments

## Abstract

**Background:**

Prostate cancer (PCa) is the most commonly diagnosed malignancy reported to Australian cancer registries with numerous studies from individual registries summarizing diagnostic and treatment characteristics. The aim of this study was to describe annual trends in clinical and treatment characteristics, and changes in surveillance practice within a large combined cohort of men with PCa in South Australia (SA) and Victoria, Australia in 2008–2013.

**Methods:**

Common data items from clinical registries in SA and Victoria were merged to develop a cross-jurisdictional dataset consisting of 13,598 men with PCa. Frequencies were used to describe these variables using the National Comprehensive Cancer Network risk of disease progression categories in 10 year age groups. A logistic regression analysis was performed to assess the impact of a number of factors (both individually and together) on the likelihood of men receiving no active treatment within twelve months of the diagnosis (i.e. managed with active surveillance/watchful waiting).

**Results:**

Trend analysis showed that over time: (1) men in SA and Victoria are being diagnosed at older age in 2013, 66.1 (SD = 9.7) years compared to 2009 (64.5 (SD = 9.7)); (2) diagnostic methods and characteristics have changed with time; and (3) types of the treatments have changed, with more men having no active treatment. The majority of men were diagnosed with Prostate-Specific Antigen (PSA) <10 ng/mL (66 %) and Grade Group < 4 (65 %). Nearly seventy percent received radical treatment within 12 months of diagnosis, while ~20 % had no active treatment. In 14 % of cases treatment was not recorded or had not commenced. Having no active treatment was strongly associated older age, lower PSA and lower Grade Group at diagnosis, and in 2013 it was offered more frequently (more than 3 times) than in 2009 (OR = 2.63, 95 % CI: 2.16–3.22).

**Conclusions:**

Findings of this study provide the first cross-jurisdictional description of PCa characteristics and management in Australia. These findings will provide benchmarking for ongoing monitoring and feedback of disease management and outcomes of PCa through the Prostate Cancer Outcomes Registry–Australia New Zealand to improve evidence-based practice.

## Background

Prostate cancer (PCa) is the most common and prevalent tumour reported to registries in Australia and overseas [1, 2]. Management of PCa is complex and depends on patient factors such as disease characteristics at diagnosis, personal preferences, existing comorbidities, and sometimes distance to treatment centres. Treatment options include radical prostatectomy, radiotherapy, brachytherapy, and hormone deprivation therapy, depending largely on grade and stage of disease at diagnosis. Chemotherapy may be provided for palliative treatment and survival benefit for late stage PCa.

Numerous hospital-based registries in Australia and overseas have been collecting information relating on men with PCa, including disease staging, risk factors, comorbidities, treatment modalities and patient reported quality of life (QOL) at various points after diagnosis or treatment [2–6]. Such registries aim not only to assess and monitor patterns and quality of care for men diagnosed with PCa, but also to eventually improve their long term outcomes.

Data extracted from both clinical and population-based registries have been utilized in numerous studies describing annual trends in the diagnosis, clinical characteristics, and factors associated with various treatment modalities, and survival trends in Australia and other countries. Feletto et al. [7] in their recent study compared the incidence and mortality rates of PCa in Australia, USA, Canada and England, and demonstrated that incidence rates in these countries are likely to be heavily influenced by prostate-specific antigen (PSA) testing, and that there was a fall in mortality that occurred too soon to be solely a result of testing. Cooperberg et al. [3] in their study described trends in primary management of low risk disease and concluded that a significant and growing number of men with low risk disease are possibly over-treated. Meng et al. [8] examined predictors of treatment modalities in patients after initial surveillance. Of the 457 men initially offered no active treatment, 188 (41 %) went on to active treatment at a median of 1.7 years after diagnosis. Baseline characteristics associated with progression to active treatment included younger age, higher level of formal education, higher PSA at presentation of the disease and higher Gleason score.

Several studies describing trends in the diagnosis of PCa, prevalence and patterns of care were also conducted in Australia [9–12]. These studies have generally been limited to individual hospitals or registries and have not examined patterns across multiple jurisdictions. The only cross-jurisdictional study undertaken in Australia had limitations as it was based on data from centralised cancer registries which do not collect detail on clinical characteristics or treatments [13].

Studies across multiple registries generally provide broader coverage and strengthen the evidence base for evaluating patterns of care and patient outcomes; and point to opportunities for improving health outcomes in Australia [14–16]. Results from multiple registries are more generalizable than those from single registries as they would be well placed to find and control for additional sources of variation and take advantage of natural policy experiments.

To date, much of the evidence that guides clinical management decisions in men with PCa in Australia has been derived from international studies. It is unclear whether clinical characteristics and treatment patterns and outcomes among Australian men are comparable to those of men in the USA or Europe, where much of the international research is based. Therefore, the main objective of the present study was to provide an overview of socio-demographic and clinical characteristics of patients with PCa, their treatment patterns and outcomes using the data combined from two clinical registries in the states of South Australia (SA) and Victoria. The secondary objective of the study was to examine and describe potential factors associated with receiving no active treatment of the disease.

## Methods

### Study population

Beginning in 2013, the Movember Foundation, a global Men’s Health Charity Organisation founded and based in Australia, funded an initiative to seek consensus for implementation of a bi-national population based prostate cancer registry – the “Prostate Cancer Outcomes Registry –Australia New and Zealand” (PCOR-ANZ) [17]. Subsequently, a research collaboration has been partnered between the University of South Australia, Monash University, Movember and the South Australian Health and Medical Research Institute to establish the Movember Prostate Cancer Health Outcomes Research Unit, aiming to improve outcomes of men with PCa. To address this unit’s goals, which also include objectives of this study, we developed a dataset, containing amalgamated records of men with PCa from the South Australian (SA) and the Victorian PCa registries [18].

The Victorian Prostate Cancer Registry (now termed the Prostate Cancer Outcomes Registry-Victoria, or PCOR-Vic), based at Monash University, was established in 2008 [19]. The registry collects data on PCa cases from 38 metropolitan and regional public and private hospitals in Victoria. The South Australian Prostate Cancer Clinical Outcomes Collaborative (SA-PCCOC) database, based in SA, was established in 1998 to include men with PCa at all major teaching and treatment hospitals in SA [5]. More recently the database has been expanded to include private treatment facilities. Currently the registry contains data on more than 11,000 patients.

A total of 13,598 records of PCa men diagnosed and consented between 2008 and 2013 in SA and Victoria were merged into the SA-Victorian PCa health outcomes research dataset [18]. This combined dataset was developed, as a forerunner to the PCOR-ANZ [17], which is currently underway, and one of the primary objectives was to demonstrate the feasibility and value of collating and amalgamating clinical data on prostate cancer treatment and outcomes across jurisdictions, by combining data from the two states in which multi-institutional clinical registries already existed.

This combined dataset contains data on patient demographics characteristics, initial diagnosis and disease staging information, PSA history, clinical examination results, treatment details, comorbidities and complications. Follow-up data are derived from the monitoring of PSA values, clinical evidence of recurrence, any further biopsy and pathology reported, as well as patient reported symptoms and QOL data.

Records of men diagnosed with histologically confirmed PCa between 2008 and 2013 were included into this study. Detailed information about the steps of data collection and SA and Victoria clinical registries is provided elsewhere [2, 9].

### Explanatory variables

Variables extracted for analysis included the year of diagnosis, patient’s age (10-year age groups) and socioeconomic status derived from residential postcodes using the Australian Bureau of Statistic’s Socioeconomic Indexes for Areas (SEIFA) [20]. PSA levels were grouped into four categories: (1) <4 ng/mL, (2) 4.01–10 ng/mL, (3) 10.01–20 ng/mL, and (5) >20 ng/mL. Five Grade Groups were used: (1) Grade Group 1, where Gleason score < =6, (2) Grade Group 2, where Gleason score = 3 + 4, (3) Grade Group 3, where Gleason score = 4 + 3, (4) Grade Group 4, where Gleason score = 8, and (5) Grade Group 5, where Gleason score > =8 [21, 22].

The National Comprehensive Cancer Network (NCCN) risk criteria for disease progression were used to classify patients into low-, intermediate- high-risk and very high risk (v.high)/metastatic disease (Table 1) [23]. Where the clinical T category was not recorded, if the Grade Group was 1 and the PSA concentration was <10 ng/mL, the patient was deemed to be at low risk for disease progression [10].

**Table 1:** Risk adjustment model adopted among men with PCa from clinical registries in SA and Victoria

Variable	NCCN
Low	Clinical T1–T2a stage AND GS 2–6 AND PSA level <10 ng/mL
Intermediate	Clinical T2b–T2c stage OR GS = 7 OR PSA level 10–20 ng/mL
High	Clinical T3a stage OR GS 8–10 OR PSA level >20 ng/mL
Very high (locally advanced)	Clinical T3b–T4
	Any T, N1
Metastatic	Any T, Any N, M1

*NCCN* National Comprehensive Cancer Network, *GS* Gleason Score, *PSA* Prostate Specific Antigen

Initial treatments within twelve months of the diagnosis were included in the analysis and classified into: (1) radical prostatectomy (RP), (2) radiotherapy (RT), (3) Androgen Deprivation Therapy (ADT), (4) active surveillance/watchful waiting (no active treatment), and (5) others (high intensity focused ultrasound, cryotherapy, chemotherapy etc.). Note that we could not reliably differentiate watchful waiting from active surveillance across the two states, and thus we have called them “no active treatment”. Where treatment information was missing (i.e. the treatment field in the registry was left blank) it was coded as ‘unknown’ rather than no active treatment. Active treatment was defined as any RP, RT, ADT or other treatment, but excluded no active treatment option. Time to the first active treatment within twelve months was calculated as a difference in days between the date of diagnosis and commencement of the first treatment. Further information about treatment types and groups is available elsewhere [18].

### Statistical analysis

Descriptive statistics were used to summarize these variables. Statistical differences in the data were assessed using the *X*^*2*^ test for categorical variables and Mann–Whitney *U*-tests for continuous variables (age and time to treatment). Since coverage was low in 2008, temporal trend analysis was undertaken from 2009.

A logistic regression analysis was performed to assess the impact of a number of factors (both individually and together) on the likelihood of men not being offered any immediate treatment (no active treatment within 12 months). In the present study, the model predicted no active treatment (i.e. “no active treatment” outcome was set as 1, and all other treatments as 0) from demographic characteristics (age group, residential area, SEIFA), and diagnostic characteristics (year of diagnosis, method of diagnosis, PSA level, Grade Group and NCCN risk). All factors were significantly predictive and were added to the multivariate model.

Statistical analyses were conducted using the Statistical Package for Social Sciences (SPSS v.22). The significance of each time trend was assessed via the Mantel-Haenszel *χ*^*2*^ test for trend. Finally, we assessed the association of sociodemographic and diagnostic variables with treatment selection, using the *χ*^*2*^or Mantel-Haenszel *χ*^*2*^ test, as appropriate. All statistical tests were conducted at the two-sided *p* < 0.05 level of significance.

## Results

### Demographic and diagnostic characteristics

A total of 13,598 men diagnosed with PCa between 2008 and 2013 in SA and Victoria were included in the analysis. The average (SD) age of study participants at diagnosis was 65.4 (9.6) years. The majority (70.5 %) of men resided in metropolitan regions (Table 2).

**Table 2:** Demographic and diagnostic characteristics among men with PCa from clinical registries in SA and Victoria

	Patients, N	%
Included into the study	13,598	100
State		
SA	3,526	25.9
Victoria	10,072	74.1
Age groups		
< 55	2,059	15.1
56–65	4,851	35.7
65–75	4,711	34.6
> 75	1,977	14.5
Age (mean, SD)	65.4 (9.6)	
Residential area		
Metropolitan	9,586	70.5
Regional/Rural	3,250	23.9
Unknown	762	5.6
SEIFA		
Lowest 10 % (0–20 %)	1,751	12.9
Lowest 21–40 %	2,245	16.5
Lowest 41–60 %	2,075	15.3
Highest 61–80 %	2,840	20.9
Highest 81–100 %	4,334	31.9
Unknown	353	2.6
Method of diagnosis		
TRUS	11,518	84.7
TURP	1,239	9.1
Other	841	6.2
PSA (ng/mL)		
< 4	2,691	19.8
4.01–10	5,985	44.0
10.01–20	1,820	13.4
> 20.01	1,284	9.4
Unknown	1,818	13.4
Grade Group		
Grade Group 1 (Gleason score ≤ 6)	4,769	35.1
Grade Group 2 (Gleason score 3 + 4)	3,771	27.7
Grade Group 3 (Gleason score 4 + 3)	1,832	13.5
Grade Group 4 (Gleason score 8)	1,264	9.3
Grade Group 5 (Gleason score >8)	1,193	8.8
Unknown	769	5.7
NCCN Risk		
Low	3,352	24.7
Intermediate	5,727	42.1
High	2,943	21.6
Very high/Metastatic	546	4.0
Unknown	1,030	7.6

*SA* South Australia, *SEIFA* Socio-Economic Index of Advantage and Disadvantage, *NCCN* National Comprehensive Cancer Network, *GS* Gleason Score, *PSA* Prostate Specific Antigen, *TURP* Transurethral Resection of the Prostate, *TRUS* Transrectal Ultrasonography of the Prostate

The majority of men (84.7 %) were diagnosed via transrectal ultrasound (TRUS) procedures and only 9.1 % by transurethral resection of prostate (TURP). Half of all men (50.8 %) with recorded PSAs at the time of diagnosis presented with PSA levels of 4.01-10 ng/mL; one third of patients (35.1 %) were diagnosed with Grade Group 1, followed by 27.7 % of men with Grade Group 2. The majority (42.1 %) of men were diagnosed with intermediate risk of disease progression.

### Treatment characteristics

Table 3 shows treatment types and time to the initial treatment stratified by the NCCN risk category. Of the men in the low NCCN risk category, nearly half (44.2 %) had no active treatment. A large proportion (34.7 %) of men in the same risk category had a RP, followed by 12.5 % of men who underwent RT. The remaining patients (0.5 %) were offered ADT or other types of treatment (4.6 %). The median [IQR] time between diagnosis and the active treatment in this group was 119 [63–222.5] days.

**Table 3:** Treatment modalities in men with PCa from clinical registries in SA and Victoria, stratified by NCCN risk group

NCCN Risk	Low*		Intermediate*		High*		V.high/Metastasis*		Total	
	N	%	N	%	N	%	N	%	N	%
RP	1,164	34.7	3,097	54.1	988	33.6	57	10.4	5,306	42.2
RT	418	12.5	1,263	22.1	958	32.6	153	28.0	2,792	22.2
ADT	18	0.5	114	2.0	469	15.9	219	40.1	820	6.5
No active treatment	1,483	44.2	723	12.6	225	7.6	18	3.3	2,449	19.5
Other	154	4.6	307	5.4	194	6.6	45	8.2	700	5.6
Unknown	115	3.4	223	3.9	109	3.7	54	9.9	501	4.0
Total	3,352	100	5,727	100	2,943	100	546	100	12,568	100
Median [IQR] days to treatment	119 [63–222.5]		80 [48–137]		49 [29–96]		31 [12–71.5]		75 [41–142]	

*NCCN* National Comprehensive Cancer Network, *RP* Radical prostatectomy, *RT* radiotherapy, *ADT* Androgen Deprivation Therapy

*p < 0.05

In the intermediate NCCN risk group a significantly higher proportion of men, relative to the low risk group, were offered an active treatment: 54.1 % of men had a RP, and 22.1 % of men were treated with RT. Only 9.4 % of men had no active treatment. The median [IQR] time between diagnosis and active treatment decreased to 80 [48–137] days.

In the high risk cancer group, 33.6 % of men had a RP and 32.6 % were treated with RT. ADT was administered in 15.6 % of men. A median [IQR] time between diagnosis and active treatment was 49 [29–96] days.

In a very high/metastasis group most of the patients (40.1 %) were treated with ADT, followed by 28.0 % of men who were offered RT. Only 10.4 % of patients had a RP. A median [IQR] time to the treatment in this risk group was significantly shorter than in other NCCN risk groups, only 31 (12–71.5) days.

### Temporal trends in demographic, diagnosis and treatment characteristics

**Fig. 1:**
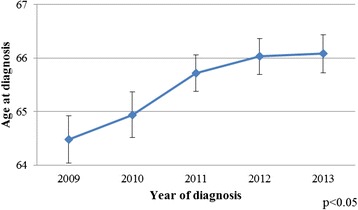
Age trends among men diagnosed with PCa from clinical registries in SA and Victoria

Annual trends of average age of men at the time of PCa diagnosis are depicted in Fig. 1, indicating that men are being diagnosed at slightly older age in 2013 (66.1 (SD = 9.7) years) when compared to 64.5 (SD = 9.7) years in 2009, *p* < 0.05.

**Fig. 2:**
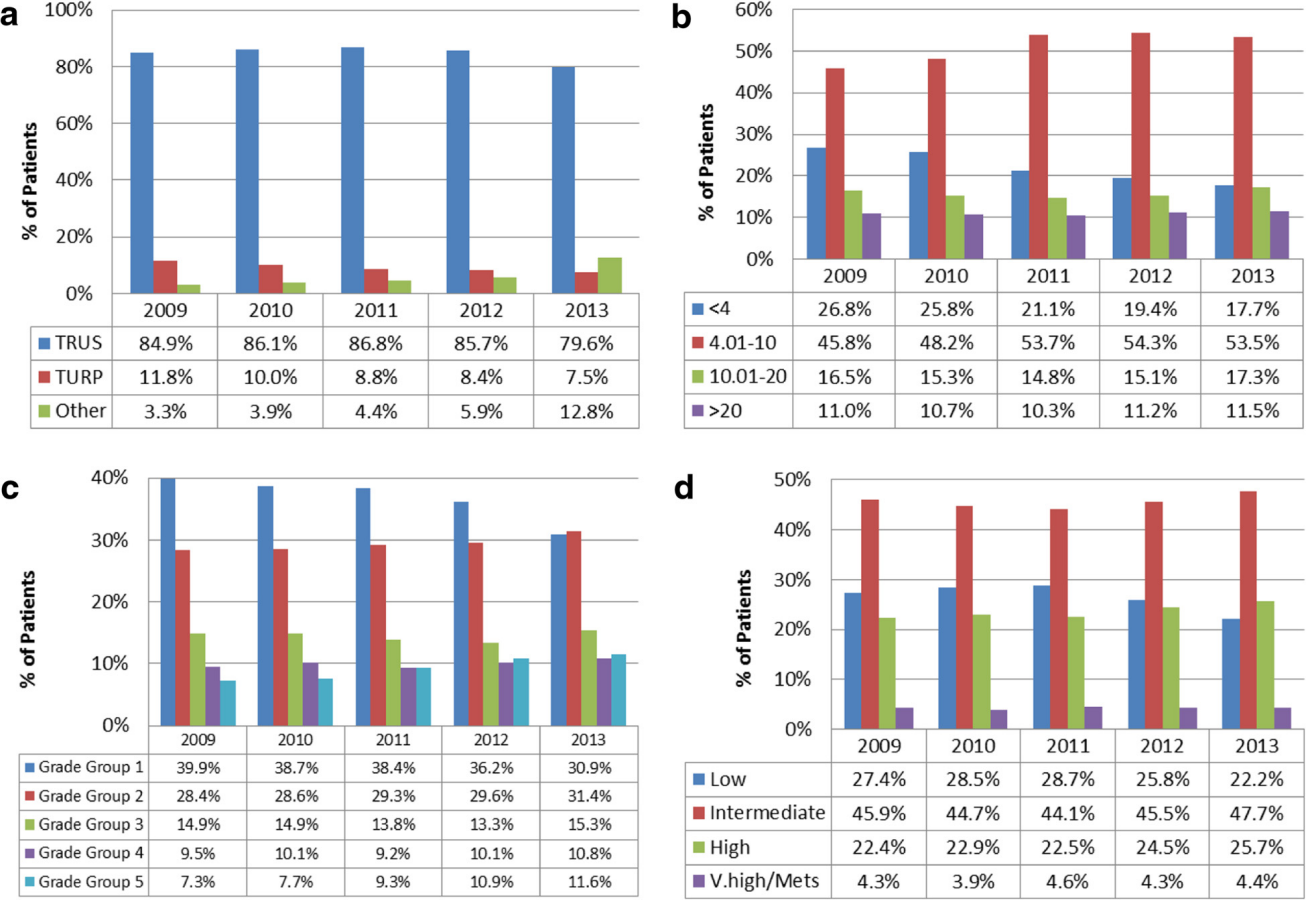
Trends in method of diagnosis (**a**), PSA levels (**b**), Grade Groups (**c**) and NCCN risk (**d**) among men diagnosed with PCa from clinical registries SA and Victoria. *p* < 0.05 for all trends, TURP, Transurethral Resection of the Prostate; TRUS, Transrectal Ultrasonography of the Prostate

Time trends in diagnostic characteristics are shown in Fig. 2. About 80 % of men were diagnosed via TRUS, and this trend remained stable from 2009–2013. A significant increase (*p* < 0.05) in the proportion of men diagnosed via “Other” diagnostic methods was noticed in 2013 (Fig. 2a).

Figure 2b summarizes temporal trends in PSA levels at diagnosis. Compared to 2009, fewer patients were diagnosed with PSA < 4.0 mL each year, while the proportion of men with PSA 4.01–10 mL increasing from 45.8 % in 2009 to 53.5 % in 2013, *p* < 0.05.

Trends in Grade Group at diagnosis are shown in Fig. 2c. The proportion of men diagnosed with Grade Group 1 reduced from 39.9 % in 2009 to 30.9 % in 2013, *p* < 0.05; while more men (31.4 %) were diagnosed with the Grade Group 2 in 2013 when compared to 28.4 % in 2009, *p* < 0.05. The proportion of men with low risk disease declined from 27.4 % in 2009 to 22.2 % in 2013, *p* < 0.05 (Fig. 2d).

**Fig. 3:**
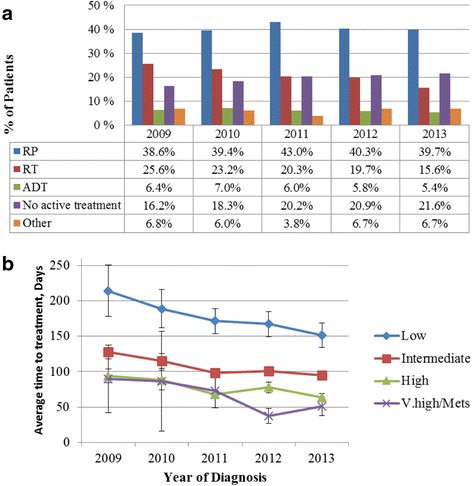
Treatment types (**a**) and time to treatment (**b**) among men diagnosed with PCa from clinical registries in SA and Victoria. *p* < 0.05 for all trends. RP – Radical Prostatectomy; RT – Radiotherapy; ADT – Androgen Deprivation Therapy

Trends in treatment modalities and time to the first treatment over the five years are shown in Fig. 3. The proportion of men with no active treatment increased from 16.2 % in 2009 to 21.6 % in 2013, *p* < 0.05 (Fig. 3a). This increase was associated with a concomitant 10 % decline in men receiving RT (from 25.6 % to 15.6 %). RP trend remained stable over the years.

Figure 3b depicts trends in duration (in days) between the diagnosis and initial active treatment across NCCN risk groups. Time interval between the diagnosis and initial treatment from 2009 to 2013 declined significantly by 62.8, 32.9, 30.3 and 39.5 days in low, intermediate, high and v.high/metastatic NCCN risk groups respectively.

### Regression analysis of factors determining surveillance of PCa

Table 4 summarizes the contributions of each factor in the univariate and multivariate model to men receiving no active treatment. Univariate analysis (step 1) for all nine categorical variables was conducted to identify factors associated with no active treatment of the disease. The nine category variables were then added into a multivariate model (step 2).

**Table 4:** Factors associated with the likelihood of no active treatment in men with PCa from clinical registries in SA and Victoria

	Univariate model		Multivariate model	
Factors	Odds Ratio	CI at 95 %	Odds Ratio	CI at 95 %
Year of Diagnosis				
2009 (Ref)	1		1	
**2010**	1.16	0.98–1.37	**1.26**	**1.02–1.55**
**2011**	**1.32**	**1.13–1.53**	**1.43**	**1.18–1.73**
**2012**	**1.37**	**1.18–1.59**	**1.82**	**1.51–2.21**
**2013**	**1.43**	**1.23–1.66**	**2.63**	**2.16–3.22**
Age Group				
< 55 (Ref)	1		1	
**56–65**	**1.29**	**1.11–1.49**	**1.48**	**1.24–1.76**
**65–75**	**1.38**	**1.19–1.59**	**2.03**	**1.69–2.44**
**> 75**	**2.01**	**1.71–2.36**	**5.83**	**4.56–7.45**
State				
SA (Ref)	1		1	
**Victoria**	**1.74**	**1.54–1.95**	**1.49**	**1.16–1.65**
Residential area				
Metropolitan (Ref)	1		1	
Regional/Rural	1.07	0.97–1.18	1.02	0.88–1.19
SEIFA				
Lowest 0–20 % (Ref)	1		1	
Lowest 21–40 %	0.96	0.81–1.14	0.85	0.69–1.07
Lowest 41–60 %	0.97	0.82–1.15	0.97	0.77–1.22
Highest 61–80 %	1.04	0.88–1.22	0.98	0.79–1.21
**Highest 81–100 %**	**1.28**	**1.11–1.48**	**1.32**	**1.07–1.63**
Method of diagnosis				
TRUS (Ref)	1		1	
**TURP**	**5.23**	**4.61–5.93**	**6.19**	**5.08–7.54**
Other	0.80	0.65–0.98	1.27	0.93–1.74
PSA (ng/mL)				
< 4 (Ref)	1		1	
**4.01–10**	**0.81**	**0.73–0.91**	1.15	0.89–1.34
**10.01–20**	**0.62**	**0.53–0.72**	**1.65**	**0.92–2.07**
**> 20.01**	**0.36**	**0.29–0.44**	1.31	0.89–1.91
Grade Group				
Grade Group 1 (Gleason score ≤ 6) [Ref]	1		1	
Grade Group 2 (Gleason score 3 + 4)	**0.19**	**0.17–0.22**	**0.33**	**0.27–0.42**
Grade Group 3 (Gleason score 4 + 3)	**0.09**	**0.07–0.12**	**0.13**	**0.09–0.17**
Grade Group 4 (Gleason score 8)	**0.06**	**0.05–0.08**	**0.08**	**0.05–0.13**
Grade Group 5 (Gleason score >8)	**0.07**	**0.06–0.09**	**0.07**	**0.05–0.12**
NCCN Risk				
Low (Ref)	1		1	
**Intermediate**	**0.17**	**0.15–0.19**	**0.35**	**0.28–0.44**
**High**	**0.09**	**0.08–0.12**	**0.23**	**0.15–0.34**
**V.high/Metastatic**	**0.02**	**0.02–0.06**	**0.06**	**0.03–0.11**

*SA* South Australia, *SEIFA* Socio-Economic Index of Advantage and Disadvantage, *NCCN* National Comprehensive Cancer Network, *GS* Gleason Score, *PSA* Prostate Specific Antigen, *TURP* Transurethral Resection of the Prostate, *TRUS* Transrectal Ultrasonography of the Prostate

Significant rows are highlighted in bold

A full multivariate model containing all nine category variables (inclusive of the variables with non-missing values within each category, year of diagnosis >2008) was statistically significant, *χ*^2^(25, *N* = 10,496) =7895,621, *p* < 0.05) indicating ability to distinguish between men with PCa who had no active treatment (*N* = 2,252) *vs* other type of treatment. The model explained between 25 % (Cox and Snell R Square) and 39 % (Nagelkerke R Square) of the variance in treatment type.

When compared to 2009, each year men were more likely to be managed with no active treatment. For example, men diagnosed in 2012 had nearly twice the odds of having no active treatment (OR = 1.82, 95 % CI, 1.51–2.21), and in 2013 even higher odds, OR = 2.63, 95 % CI, 2.16–3.22). Men older than 75 years of age had nearly three times the odds of receiving no active treatment, compared to younger men of 55 years or less, OR = 5.83, (95 % CI, 4.56–7.45). Men with PCa were also more likely not to receive an active treatment in Vic, OR = 1.49, (95 % CI, 1.165–1.65). Men in the highest 81–100 % quintile of SEIFA were significantly more likely to have no active treatment (OR = 1.32, 95 % CI, 1.07–1.63), compared to those in the lowest (0–20 %) quintile of SEIFA.

Those men whose diagnosis was detected via TURP were more likely to not to receive an active treatment, OR = 6.19, (95 % CI, 5.08–7.54) than men diagnosed via TRUS. Men diagnosed with higher Grade Group were significantly less likely to be offered an active treatment. For example, men with Grade Group 5 had a 93 % lower odds of receiving no active treatment than men diagnosed with Grade Group 1, OR = 0.07, (95 % CI, 0.05–0.12). Similarly, men in higher NCCN risk groups were more likely to be offered an active treatment, when compared to those in a low risk category.

## Discussion

### General findings

To our knowledge, this was the first large-scale retrospective population-based cohort study for which authors accessed the data records from multiple clinical registries of men diagnosed with PCa in Australia. The major findings of this study indicate that in the 2008–13 period: (1) men are being diagnosed at older age; (2) diagnostic methods and characteristics have changed and (3) types of the treatments have changed, with more men in lower risk groups being offered no active treatment, and primary radiation treatment becoming less frequent.

### Comparison with the existing literature

Consistent with the findings of previous studies, the average age at diagnosis of the men in our cohort was 65 years [3, 7, 24]. However, we also observed that over the period of five years, age at the diagnosis has slightly increased. This could be due to the recent decline in PSA testing among Australian men, which in turn may be leading to men being diagnosed at an older age and with a higher PSA [25, 26]. Consistent with this is the decrease in proportion of men diagnosed with PSA less than 4 ng/mL. Alternatively, younger men with low PSAs may not have been biopsied as frequently in 2013 compared with 2008.

Nearly half of all men were diagnosed with PSA levels of <10 mL. This became a constant trend in 2011, which could possibly be explained by the increasing use of PSA blood tests in case-finding from 1990–2010, resulting in the decreased proportion of PCa patients with high PSA levels [27]. Our findings are similar to those with Galan et al. [28], who showed that tumours currently detected tend to appear with lower PSA levels, and localized clinical stages. A similar trend was also observed in decreasing rates of high grade cancer, denoted by the Grade Group. The proportion of men with Grade Group 1 in 2013 declined by 10 % when compared to 2009.

TRUS remains the most commonly used PCa detection method with stable trends over the years. However, an increasing percentage of other diagnostic tools in 2013 suggest that more advanced diagnostic and investigation/staging techniques such as transperineal prostate biopsy [29] or multiparametric magnetic resonance (mMRI), that are becoming more widely used in Australia. mMRI is emerging as a useful tool in the investigation and treatment of PCa, by identifying regions which may represent clinically significant PCa [30–32].

RP and RT were the most commonly offered treatment types to men with PCa in SA and Victoria [10, 33]. However, recently the proportion of men undertaking RT treatment has declined while numbers of those with no active treatment have increased. Notably, the proportion of men treated with RP did not materially change over the years, but the higher proportion of men managed with no active treatment over the time is matched with the lower proportion of men managed with initial RT. No active treatment is usually recommended for patients with low risk disease, older men and where active treatment might be more harmful rather beneficial. Our findings are similar to those of the USA and European studies, where more men are opting for this conservative management in [34, 35].

We have also demonstrated that lower risk disease, older age at diagnosis, lower PSA levels and Grade Group were factors strongly associated with the conservative management of the disease, as have others [3, 8]. Differences between the two states may be due in part to idiosyncrasies in the way surveillance is recorded in each registry. The trend toward increased use of no active treatment indicates the increasing prominence of Prostate Cancer Research International: Active Surveillance guidelines which encourage clinicians to avoid active treatment in cases where risk of progression is considered to be low [36, 37]. Clinical registries may also play an important role in that reporting back to clinicians might have impacted on the management path of men with low risk disease [38]. Hamilton et al. [39], in a study of seven registries in the USA, made the distinction between men receiving no therapy with no monitoring plan (no therapy/no plan [NT/NP]) and those under active surveillance or (i.e. having no active treatment) with proposed delayed active intervention. The study found that physician and clinical factors were stronger predictors of active surveillance, whereas demographic and regional factors were related to NT/NP. Older age at diagnosis, lower clinical risk group, and geographic location were significant predictors of use of both active surveillance and NT/NP. Physicians appeared reluctant to recommend no active treatment for younger patients with no comorbidities. Loeb et al. [40] have reported that - since 2007, 59 %, 41 % and 16 % of men in Sweden with very low, low and intermediate risk PCa, respectively, were under active surveillance and watchful waiting (i.e. had no active treatment) rather had active treatment. Age was by far the strongest determinant of receiving no active treatment. Education, marital status and comorbidity were significantly but weakly associated with deferring treatment.

### Study limitations and strengths

The major strength of this study is the use of clinical registries, containing a detailed diagnosis and treatment information of patients with PCa in SA and Victoria. These registries enable rapid and reliable ascertainment of patterns-of-care of patients and up-to-date reporting back to treating clinicians [2]. However, limitations need to be noted as well.

Firstly, treatment classification was slightly different across states, such that we were unable to accurately determine the intent of observation (i.e. whether under active surveillance with intent to curatively treat if disease progressed, or watchful waiting with palliative treatment offered if necessary). Therefore these two modalities were combined into one group called “no active treatment”.

Secondly, we were unable to assess and describe trends in type of hospital where patients were treated as the information in both registries was different. For example, the type of hospital where a patient was treated in Victoria was coded as “private” or “public” depending on the hospital type; however in SA patients are classified as being either “public” or “private” rather than that descriptor relating to the health care facility [18]. Treatment type information was missing or unknown in ~14 % of cases. We were unable to assess the impact of comorbidities such as chronic illness and obesity on patterns of disease management as such information is not collected in either registry. In addition, neither state had 100 % population coverage of PCa cases.

## Conclusions

This was the first study to describe patterns of care and trends in diagnostic characteristics in men with PCa across two registries in Australia. The recently developed PCOR-ANZ will collect patterns of care and standardised patient reported QOL measures of men nation-wide in Australia and New Zealand [17]. This information will be incorporated into future analyses to be conducted and will assist in transforming healthcare for men with PCa in Australia and New Zealand by encouraging change in practice in line with guidelines/recommendations (e.g. offering active surveillance in low risk disease and observation for older men with less life expectancy) through monitoring and reporting outcomes and feedback to clinicians caring for men with PCa.

## Abbreviations

ADT, Androgen Deprivation Therapy; IQR, interquartile range; mMRI, multiparametric magnetic resonance imaging; NCCN, National Comprehensive Cancer Network; NT/NP, no therapy/no plan; OR, odds ratio; PCa, prostate cancer; PCOR-ANZ, Australian and New Zealand Prostate Cancer Outcomes Registry; PCOR-Vic, Prostate Cancer Outcomes Registry – Victoria; PSA, prostate-specific antigen; QOL, quality of life; RP, radical prostatectomy; RT, radiation therapy; SA, South Australia; SAHMRI, South Australian Health and Medical Research Institute; SA-PCCOC, South Australian Prostate Cancer Clinical Outcomes Collaborative; SD, standard deviation; SEIFA, socio-economic index of advantage and disadvantage; TRUS, transrectal ultrasonography of the prostate; TURP, transurethral resection of the prostate; Vic, Victoria

## References

[1] Center M, Jemal A, Lortet-Tieulent J, Ward E, Ferlay J, Brawley O, Bray F. International variation in prostate cancer incidence and mortality rates. Eur Urol 2012, 61(6):doi: 10.1016/j.eururo.2012.1002.1054. Epub 2012 Mar 1018. 10.1016/j.eururo.2012.02.054 22424666

[2] Evans S, Millar J, Wood J, Davis I, Bolton D, Giles G, Frydenberg M, Frauman A, Costello A, McNeil J (2012). The prostate cancer registry: monitoring paterns and quality of care for men diagnosed with prostate cancer. BJU Int.

[3] Cooperberg MR, Broering JM, Litwin MS, Lubeck DP, Mehta SS, Henning JM, Carroll PR (2004). The contemporary management of prostate cancer in the United States: Lessons from the cancer of the prostate strategic urologic research endeavor (capsure), a national disease registry. J Urol.

[4] Gandaglia G, Bray F, Cooperberg MR, Karnes RJ, Leveridge MJ, Moretti K, Murphy DG, Penson DF, Miller DC. Prostate cancer registries: Current status and future directions. Eur Urol. 2016;69(6):998–1012. doi:10.1016/j.eururo.2015.05.046. Epub 2015 Jun 6. 10.1016/j.eururo.2015.05.046 26056070

[5] South Australian Prostate Cancer Clinical Outcome Collaborative [http://www.sa-pccoc.com]; 1998.

[6] Schmidt S, Garin O, Pardo Y, Valderas J, Alonso J, Rebollo P, Rajmil L, Garcia-Forero C, Ferrer F. Assessing quality of life in patients with prostate cancer: A systematic and standardized comparison of available instruments. Qual Life Res 2014. doi:10.1007/s11136-014-0678-8. 10.1007/s11136-014-0678-8 PMC4155169 24748557

[7] Feletto E, Bang A, Cole-Clark D, Chalasani V, Rasiah K, Smith DP. An examination of prostate cancer trends in Australia, England, Canada and USA: Is the australian death rate too high? World J Urol. 2015;33(11):1677–87. doi:10.1007/s00345-015-1514-7. Epub 2015 Feb 20 10.1007/s00345-015-1514-7 PMC4617845 25698456

[8] Meng MV, Elkin EP, Harlan SR, Mehta SS, Lubeck DP, Carroll PR (2003). Predictors of treatment after initial surveillance in men with prostate cancer: results from capsure. J Urol.

[9] Beckmann K, Pinnock C, Tamblyn D, Kopsaftis T, AMF S, Roder D (2009). Clinical and socio-demographic profile of an australian multi-institutional prostate cancer cohort. Clin Oncol.

[10] Evans S, Millar J, Davis C, Murphy D, Bolton D, Giles G, Frydenberg M, Andrianopoulos N, Wopod J, Frauman A (2013). Patterns of care for men diagnosed with prostate cancer in Victoria from 2008 to 2011. Med J Aust.

[11] Evans S, Millar J, Frydenberg M, Murphy D, Davis C, Spelman T, Bolton D, Giles G, Dean J, Costello A et al. Positive surgical margins: Rate, contributing factors and impact on further treatment: Findings from the prostate cancer registry. BJU Int 2013, doi: 10.1111/bju.12509 10.1111/bju.12509 24128010

[12] Smith D, King M, Egger S, Berry M, Stricker P, P C, Ward J, DL OC, Armstrong B. Quality of life three years after diagnosis of localised prostate cancer: Population based cohort study. BMJ. 2009;339:b4817. doi:10.1136/bmj.b4817. 10.1136/bmj.b4817 PMC2784818 19945997

[13] Baade PD, Youlden DR, Coory MD, Gardiner RA, Chambers SK (2011). Urban–rural differences in prostate cancer outcomes in Australia: what has changed?. Med J Aust.

[14] Adami H, Bergstrom R, Mohner M, Zatosnki W, Storm H, Ekbom A, Tretli S, Teppo L, Ziegler H, Rahu M (1994). Testicular cancer in nine northern european countries. Int J Cancer.

[15] Coleman M, Forman D, Bryant H, Butler J, Rachet B, Maringe C, Nur U, Tracey E, Coory M, Hatcher J (2011). Cancer survival in Australia, Canada, Denmark, Norway, Sweden, and the UK, 1995–2007 (the international cancer benchmarking partnership): an analysis of population-based cancer registry data. Lancet.

[16] Verdecchia A, Francisci S, Brenner H, Gatta G, Micheli A, Mangone L, Kunkler I (2007). Recent cancer survival in Europe: a 2000–02 period analysis of EUROCARE-4 data. Lancet.

[17] Evans S, Nag N, Roder D, Brooks A, Millar J, Moretti K, Pryor D, Skala M, McNeil J. Development of the international Prostate Cancer Outcomes Registry. BJU Int 2015, doi: 10.1111/bju.13258 10.1111/bju.13258 26877056

[18] Ruseckaite R, Beckmann K, O’Callaghan M, Roder D, Moretti K, Zalcberg J, Millar J, Evans S (2016). Development of South Australian-Victorian Prostate Cancer Health Outcomes Research Dataset (SA-VIC PCHORD). BMC Res Notes.

[19] Victorian Prostate Cancer Clinical Registry (PCR). 2008. http://pcr.registry.org.au. Accessed Nov 2015.

[20] Australian Bureau of Statistics. 2015. www.abs.gov.au. Accessed Nov 2015.

[21] Epstein JI, Zelefsky MJ, Sjoberg DD, Nelson JB, Egevad L, Magi-Galluzzi C, Vickers AJ, Parwani AV, Reuter VE, Fine SW et al. A contemporary prostate cancer grading system: A validated alternative to the gleason score. Eur Urol. 2016;69(3):428–35. doi:10.1016/j.eururo.2015.06.046. Epub 2015 Jul 10. 10.1016/j.eururo.2015.06.046 PMC5002992 26166626

[22] Epstein JI, Egevad L, Amin MB, Delahunt B, Srigley JR, Humphrey PA (2016). The 2014 international society of urological pathology (ISUP) consensus conference on Gleason grading of prostatic carcinoma: definition of grading patterns and proposal for a new grading system. Am J Surg Pathol.

[23] Prostate cancer staging. Https://cancerstaging.Org/references-tools/quickreferences/documents/prostatesmall.Pdf 2015.

[24] Boscoe FP, Pradhan E (2015). A medicare-associated spike in U.S. Cancer rates at age 65, 2000–2010. Public Health Rep.

[25] O’Kelly F, Thomas A, Murray D, Galvin D, Mulvin D, Quinlan DM (2013). Can delayed time to referral to a tertiary level urologist with an abnormal PSA level affect subsequent gleason grade in the opportunistically screened population?. Prostate.

[26] Roumiguie M, Beauval JB, Bordier B, Filleron T, Rozet F, Ruffion A, Mottet N, Cussenot O, Malavaud B. What risk of prostate cancer led urologist to recommend prostate biopsies? Prog Urol. 2015;25(16):1125–31. doi:10.1016/j.purol.2015.08.007. Epub 2015 Oct 1. 10.1016/j.purol.2015.08.007 26431746

[27] Kitagawa Y, Machioka K, Yaegashi H, Nakashima K, Ofude M, Izumi K, Ueno S, Kadono Y, Konaka H, Mizokami A (2014). Decreasing trend in prostate cancer with high serum prostate-specific antigen levels detected at first prostate-specific antigen-based population screening in Japan. Asian J Androl.

[28] Lujan Galan M, Paez Borda A, Chiva Robles V, Santonja Garriga C, Romero Cajigal I, Berenguer Sanchez A (2004). Epidemiological trends in prostate cancer over the last years. Arch Esp Urol.

[29] Dimmen M, Vlatkovic L, Hole KH, Nesland JM, Brennhovd B, Axcrona K (2012). Transperineal prostate biopsy detects significant cancer in patients with elevated prostate-specific antigen (PSA) levels and previous negative transrectal biopsies. BJU Int.

[30] Pedler K, Kitzing YX, Varol C, Arianayagam M (2015). The current status of MRI in prostate cancer. Aust Fam Physician.

[31] Scheenen TW, Rosenkrantz AB, Haider MA, Futterer JJ (2015). Multiparametric magnetic resonance imaging in prostate cancer management: Current status and future perspectives. Invest Radiol.

[32] Katelaris NC, Bolton DM, Weerakoon M, Toner L, Katelaris PM, Lawrentschuk N (2015). Current role of multiparametric magnetic resonance imaging in the management of prostate cancer. Korean J Urol.

[33] Frydenberg M (2014). Prostate cancer: care beyond prostate cancer-improving patient outcomes. Nat Rev Urol.

[34] McCarthy M (2015). More US men with low risk prostate cancer opt for watchful waiting. BMJ.

[35] Hager B, Kraywinkel K, Keck B, Katalinic A, Meyer M, Zeissig SR, Stabenow R, Froehner M, Huber J (2015). Integrated prostate cancer centers might cause an overutilization of radiotherapy for low-risk prostate cancer: a comparison of treatment trends in the united states and Germany from 2004 to 2011. Radiother Oncol.

[36] Bangma CH, Bul M, Roobol M (2012). The prostate cancer research international: active surveillance study. Curr Opin Urol.

[37] Bul M, Zhu X, Valdagni R, Pickles T, Kakehi Y, Rannikko A, Bjartell A, van der Schoot DK, Cornel EB, Conti GN (2013). Active surveillance for low-risk prostate cancer worldwide: The PRIAS study. Eur Urol.

[38] Weerakoon M, Papa N, Lawrentschuk N, Evans S, Millar J, Frydenberg M, Bolton D, Murphy DG (2015). The current use of active surveillance in an Australian cohort of men: a pattern of care analysis from the Victorian prostate cancer registry. BJU Int.

[39] Hamilton AS, Wu XC, Lipscomb J, Fleming ST, Lo M, Wang D, Goodman M, Ho A, Owen JB, Rao C (2012). Regional, provider, and economic factors associated with the choice of active surveillance in the treatment of men with localized prostate cancer. J Natl Cancer Inst Monogr.

[40] Loeb S, Berglund A, Stattin P (2013). Population based study of use and determinants of active surveillance and watchful waiting for low and intermediate risk prostate cancer. J Urol.

